# Integrative transcriptomic analysis identifies immune-associated candidate genes and altered immune cell infiltration in pulmonary arterial hypertension

**DOI:** 10.1371/journal.pone.0350015

**Published:** 2026-05-22

**Authors:** Xitong Yang, Bin Zhou, Ying Yang, Yu Dong, Jifen Fu, Hong Liu, Xinhua Wu

**Affiliations:** 1 Faculty of Life Science and Technology, Kunming University of Science and Technology, Kunming, Yunnan, China; 2 Medical school, Kunming University of Science and Technology, Kunming, Yunnan, China; 3 The First Affiliated hospital of Dali University, Dali, Yunnan, China; Tokyo Women's Medical University, JAPAN

## Abstract

**Background:**

Pulmonary arterial hypertension (PAH) is a progressive vascular disease characterized by immune dysregulation and pulmonary vascular remodeling. This study aimed to identify immune-associated hub genes in PAH using an integrative bioinformatics framework and to validate key candidates in an experimental model.

**Methods:**

Three PAH lung transcriptomic datasets from the Gene Expression Omnibus (GEO) database were analyzed. Immune cell infiltration was estimated using single-sample gene set enrichment analysis (ssGSEA). Differentially expressed genes (DEGs) were identified and integrated through weighted gene co-expression network analysis (WGCNA) and protein-protein interaction (PPI) network construction. Hub genes were prioritized using multiple machine learning algorithms. A PAH-relevant murine model (Su5416 combined with hypoxia) was used for *in-vivo* validation by quantitative real-time PCR.

**Results:**

A total of 8 hub genes were identified through integrative screening across multiple algorithms and were validated in independent datasets. Among these hub genes, BCLAF1 demonstrated the highest diagnostic performance. Immune infiltration analysis revealed significant alterations in T helper cell subsets in PAH. Correlation analysis indicated associations between hub genes and specific immune signatures, including positive correlations of CDC5L and RBM39 with Tgd cells, a negative correlation of ASH1L with neutrophils, and inverse associations of CTNNB1 and SMARCA5 with dendritic cells (DCs) and central memory T cell (Tcm) signatures. In the PAH murine model, BCLAF1, CDC5L, SMARCA5, and ASH1L were significantly upregulated in lung tissues, accompanied by enhanced collagen deposition.

**Conclusion:**

This study identified BCLAF1, CDC5L, SMARCA5, and ASH1L as immune-associated hub genes in PAH and proposed a transcriptomic gene–immune prioritization framework. These candidates warrant further mechanistic investigation for their potential roles in PAH pathogenesis.

## 1. Introduction

Pulmonary arterial hypertension (PAH) is a progressive vasculopathy characterized by obliterative remodeling of the distal pulmonary arteries, which increases pulmonary vascular resistance and can ultimately lead to right ventricular failure and premature death [1]. Current international guidelines outline diagnostic algorithms and risk-stratified treatment strategies for PAH. However, although therapies targeting the endothelin, nitric oxide, and prostacyclin pathways can improve symptoms and outcomes, effective disease modification remains limited for many patients [2,3]. A revised hemodynamic definition has sharpened case recognition, this revision also highlights the biological heterogeneity of PAH. Although patients share a common hemodynamic and vascular phenotype, the underlying pathogenic mechanisms may differ substantially, which complicates early diagnosis and targeted treatment [2–4]. Therefore, it remains important to clarify which molecular changes drive the transition from vascular injury to persistent remodeling, and whether these changes can be exploited as diagnostic markers or therapeutic targets.

The understanding of PAH has expanded beyond traditional views of endothelial and smooth-muscle dysfunction [5]. Recent studies suggest that PAH has a strong immunological component. Abnormal innate and adaptive immune responses can aggravate endothelial injury, disturb vascular repair, and promote perivascular inflammatory remodeling [6]. In research, disruption of immune regulation [such as reduced regulatory T-cell (Treg) activity] can trigger endothelial injury [7]. This, in turn, drives inflammation and changes in blood vessel structure, suggesting that loss of immune control may play a direct causal role in PAH, rather than being an incidental finding [8]. High-resolution transcriptomic studies of human PAH lungs have revealed cell-type-specific stress responses and remodeling states across the vascular wall [9]. In parallel, single-cell analyses of pulmonary artery endothelial cells have further uncovered marked heterogeneity, potentially conferring differential susceptibility to apoptosis, inflammatory signaling, and metabolic rewiring [10,11]. Although single-cell studies in experimental PAH have improved our understanding of endothelial phenotypic transitions and intercellular signaling, these findings have not yet been translated into robust gene signatures with diagnostic value [12].

Such integrative approach is important because gene regulation in PAH reflects the combined effects of inherited susceptibility, epigenetic changes, and disease-context signaling, which together promote proliferative, apoptosis-resistant, and inflammatory vascular phenotypes [13,14]. Bromodomain-containing protein 4 (BRD4) has emerged as an important upstream regulator of pathogenic cellular responses in PAH; its inhibition can reverse established disease in preclinical models, reinforcing the rationale for discovering targets governed by chromatin-centred control mechanisms [15]. In parallel, Wnt signalling regulates pulmonary vascular homeostasis. Disruption of this pathway modulates smooth- muscle proliferation and remodelling, particularly through β-catenin-linked transcriptional output, making it a candidate therapeutic target in PAH [16–18]. Analyses of peripheral blood transcriptomes suggest that circulating gene-expression patterns may capture some features of PAH, but these signatures still require validation across independent cohorts, and their biological relevance remains to be clarified [19]. In particular, few studies have systematically integrated immune-cell infiltration analysis, network-based hub-gene prioritization, and experimental validation across multiple PAH cohorts.

**Fig 1:**
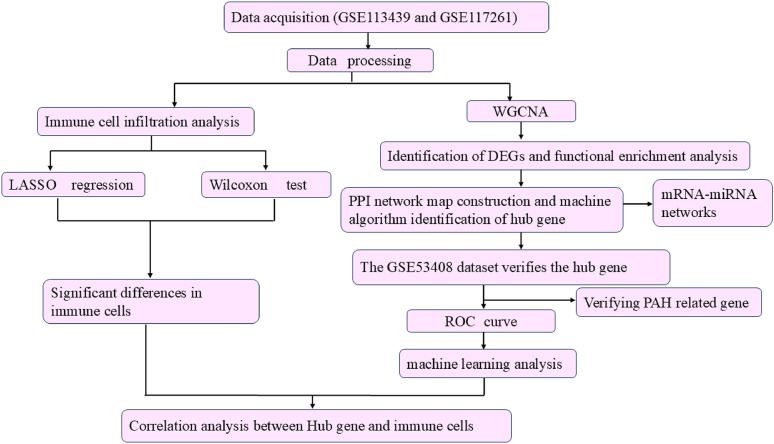
The flowchart of this study.

In this study, we used an integrative analytical strategy to identify immune-related candidate genes in PAH. Specifically, we combined immune-cell analysis, weighted gene co-expression network analysis (WGCNA), candidate-gene prioritization, and machine learning to identify immune-related candidate genes associated with PAH. Furthermore, key candidates were validated in a PAH-relevant *in-vivo* model, linking computational findings to biological evidence. The analytical workflow of this study is illustrated in Fig 1.

## 2. Materials and methods

### 2.1 Data sources

PAH and normal lung tissue microarray data were downloaded from the Gene Expression Omnibus (http://www.ncbi.nlm. nih.gov/geo/). Specific inclusion criteria were applied to ensure data quality and relevance. Available datasets required mRNA expression profiling obtained by microarray and included both PAH and normal lung tissue samples. GSE113439 and GSE117261 were selected as test sets; GSE53408 served as validation data. Empty or duplicated gene symbols were processed on the XIANTAO platform (https://www.xiantaozi.com). Dataset information is presented in Table 1.

**Table 1:** Basic information of the GSE113439, GSE117261, and GSE53408 datasets.

Characteristics	GSE113439	GSE117261	GSE53408
Platform	GPL6244	GPL6244	GPL6244
Sample source	Biopsy	Biopsy	Biopsy
PAH（n）	n = 15	n = 58	n = 12
Normal (n)	n = 11	n = 25	n = 11
Attribute	Test	Test	Verification

### 2.2 Data processing

Raw data for GSE113439 and GSE117261 were downloaded from the GEO database. Specifically, the R package “affy” (version 3.6.3) was employed to extract and normalize the expression matrix. Subsequently, the matrix of probe expressions was converted into a gene expression matrix using the platform’s annotation file. When multiple probes corresponded to a single gene, the gene expression value was calculated as the average of the corresponding probe intensities. In contrast, probes that mapped to more than one gene were excluded from this calculation. Next, batch effects were adjusted using the “ComBat” function from the R package “sva”. Finally, Uniform Manifold Approximation and Projection (UMAP) analysis was conducted utilizing the “umap” package of R, and the findings were illustrated using the R package “ggplot2.”

### 2.3 Analysis of immune cell infiltration

Immune cell infiltration in PAH and healthy lung tissues was quantified by single-sample gene set enrichment analysis (ssGSEA). Wilcoxon test and least absolute shrinkage and selection operator (LASSO) regression analysis were performed using the “glmnet” package for the regression analysis to identify immune cells with notable differences between the two groups (*p* < 0.05). Results were visualized using the “ggplot2” and “glmnet” packages.

### 2.4 WGCNA

WGCNA can be used to screen biomarkers by constructing gene co-expression networks and correlating gene modules with clinical traits [20]. Briefly, outliers were first excluded through hierarchical clustering, and a scale-free network was constructed by screening the soft threshold. Then, an adjacency matrix was constructed based on Pearson correlation coefficients between gene pairs in the network and subsequently converted into a topological overlap matrix (TOM). A hierarchical clustering tree was drawn, and genes were divided into different modules using hierarchical clustering and dynamic tree cutting functions. The module with the highest correlation coefficient (*p* < 0.05) was selected as the core module.

### 2.5 Identification of differentially expressed genes (DEGs) and functional enrichment assessment

In this study, DEGs between PAH tissues and normal lung tissues were identified using the R package “limma”, with the thresholds set at |log_2_ fold change (log_2_FC)| > 1 and adjusted *p* < 0.05. The results were visualized as heatmaps and volcano plots using the R packages “ComplexHeatmap” and “ggplot2”. For functional enrichment analysis, gene IDs were converted using the R package “org.Hs.e.g.,db”, and Gene Ontology (GO) enrichment analysis was performed with the R package “clusterProfiler”. Significantly enriched terms were defined as those with adjusted *p* < 0.05 and q-value< 0.2. The enrichment results were visualized using “ggplot2”.

### 2.6 Formation of protein-protein interaction (PPI) network, and the identification and confirmation of hub genes

PPI networks for the DEGs were constructed using the STRING online platform (https://string-db.org), with an interaction score ＞ 0.4 as the threshold. The intersection data were then imported into Cytoscape (version 3.9. 1). CytoHubba plugin with seven algorithms [Maximal Clique Centrality (MCC), Density of Maximum Neighborhood Component (DMNC), Maximum Neighborhood Component (MNC), Degree, Edge Percolated Component (EPC), BottleNeck, and EcCentricity] were used to prioritize hub genes, with each algorithm selecting the top 60 genes. The results were intersected using the R package UpSet. Significant gene modules were identified through Molecular Complex Detection (MCODE) application (degree cutoff = 2, node score cutoff = 0.2, core = 2, and depth = 100). The identified hub genes were validated using the GSE53408 dataset.

### 2.7 Prediction of miRNA targets

miRNA targets of eight hub genes were predicted using four online miRNA databases: TargetScan (version 8.0), miRWalk (version 3.0), miRtarBase (version 8.0), and miRDB (version 6.0). The miRNAs predicted by at least three databases were selected for analysis. Co-expression networks were constructed and visualized in Cytoscape.

### 2.8 Examination of the diagnostic validity of biomarkers

The diagnostic performance of hub genes was evaluated in the GSE53408 dataset. A logistic regression model was constructed employing the “glm” function from the R package. Receiver Operating Characteristic (ROC) analysis was performed using the “pROC” package, and the area under the curve (AUC) was calculated to assess diagnostic value. ROC curves were visualized using “ggplot2”.

### 2.9 Hub gene machine learning analysis

To prioritize hub genes, we applied ten classic machine learning algorithms [Least Absolute Shrinkage and Selection Operator (LASSO), Support Vector Machines (SVM), Random Forest (RF), Gradient Boosting with Component-wise Linear Models (glmBoost), Stepwise Generalized Linear Model (Stepglm), Ridge, Elastic Net (Enet), Gradient Boosting Machine (GBM), Linear Discriminant Analysis (LDA), eXtreme Gradient Boosting (XGBoost), NaiveBayes] to the training set expression data, generating 113 prediction models. Hyperparameters were optimized through 5-fold cross-validation, and stratified sampling was used to divide the data into training and validation sets. Model performance was strictly evaluated by AUC, accuracy, and F1 score. Subsequently, a stacking ensemble learning strategy was used to integrate optimal single model prediction results. High-confidence models were selected, and their feature genes were ranked according to frequency to identify core genes. Finally, the gene expression patterns were visualized using the pheatmap package.

### 2.10 Model explanation

The SHapley Additive exPlanations (SHAP) method was adopted to explain the machine learning models. This method quantified the importance of each feature by calculating the average marginal contribution of each feature across all possible model sub-combinations. Global feature ranking was determined based on the average absolute SHAP value [21].

### 2.11 Examination of relationships between hub genes and immune cells

The relationships between 8 hub genes and immune cells were analyzed using Spearman’s rank Correlation analysis utilizing the R software. Results were visualized with the “ggplot2” package in R.

### 2.12 Construction of PAH model

A total of 10 C57BL/6J mice (8 weeks, 20-25g) were purchased from Kunming Medical University and randomly grouped: normoxia control group and PAH model group (n = 5 each group). Briefly, all mice routine feeding, kept at 20−26°C with 40−70% relative Humidity, with a 12-hour light-dark cycle. The PAH mice model was established using the Sugen 5416 combined with hypoxia (SuHx) method [22]. In short, mice were placed in a hypobaric hypoxia chamber simulating 5000 m altitude and continuously exposed to hypoxic conditions (10% O_2_, 90% N_2_). Su5416 was administered once a week at 20 mg·kg ⁻ ¹ for 4 weeks [23–25]. The body weight and activity behavior of the mice were monitored daily. After modeling, all experimental animals were humanely euthanized using isoflurane anesthesia followed by cervical dislocation in accordance with American Veterinary Medical Association guidelines to minimize suffering. All procedures were approved by the Animal Ethics Committee of Dali University (2024-PZ-160) under licenses SCXK (Dian) K2020-0004 and SYXK (Dian) K2020-0006. All the experimenters have participated in the animal experiment training at Dali University.

### 2.13 Measurement of hemodynamics

All mice underwent hemodynamic assessment by Doppler echocardiography at predefined post-modelling time points using an HP/SONOS 5500 system with a 4–10 MHz transducer. Following anesthesia induction with 3% isoflurane, mice were positioned supine, with the thoracic area depilated. Next, the probe was placed on the left precordium to optimize visualization of cardiac anatomy and flow profiles. Cardiac chambers, valves, and major vessels were examined using combined M-mode and two-dimensional imaging. After that, the mean pulmonary artery pressure (mPAP) was determined. Additionally, right ventricular systolic pressure (RVSP) was estimated from the peak velocity of tricuspid regurgitation using standard Doppler-derived calculations. Furthermore, the right ventricular hypertrophy index (RVHI) was assessed using the Fulton index, calculated as the ratio of right ventricular (RV) weight to the combined weight of the left ventricle (LV) and interventricular septum [RV/(LV + S)] [26].

### 2.14 Quantitative reverse transcription-polymerase chain reaction (qRT-PCR)

Total RNA was extracted using TRIzol reagent. Next, complementary DNA (cDNA) was prepared through reverse transcription using the first-strand cDNA synthesis kit (TIANGEN, Beijing, China) according to the kit’s protocol. qRT-PCR was conducted utilizing the CFX96 Real-Time PCR System with a detection kit (Vazyme, Nanjing, China, Q711-02). The application included 40 cycles: pre-denaturation (95°C for 1 min); denaturation (95°C for 20 s); annealing (55°C for 20 s), and extension (72°C for 30 s). Relative gene expression was calculated using the 2-^ΔΔCt^ method, with β-actin as the internal control. All reactions were performed in triplicate. Primer sequences are listed in Table 2.

**Table 2:** qRT-PCR primer sequence.

Gene	Primer	Primer sequence (5′-3′)
RBM39	forward	CATGCTTGAGGCCCCTTACAA
	reverse	TTCCGCTCTCGACTTTTGCTC
BCLAF1	forward	CCCTTCTCAGCATTCACATTCC
	reverse	TCGACTCGACCCATTTCCAAC
CDC5L	forward	CCTGACCCGATAGACATGGAT
	reverse	AGCCTTCTTTCCTTGAGTATTGG
CTNNB1	forward	CCCAGTCCTTCACGCAAGAG
	reverse	CATCTAGCGTCTCAGGGAACA
SMARCA5	forward	AAAAATGCAAACTGACCGAGC
	reverse	CCCTGGTTTCATCTTCAAGGGT
CHD8	forward	GCCAAGGGAATCCTTTCATGG
	reverse	TCGAAGGGGTGTACCAGTCAG
ASH1L	forward	TTAGGATTGGGTTCTGATTCCGA
	reverse	CGATTCCGCTTGCGAGGAT
KDM6A	forward	GGAGGTGCTTTATGTCGATCC
	reverse	AAGTGGGCAATGTGAAACTGA
β-actin	forward	GGCTGTATTCCCCTCCATCG
	reverse	CCAGTTGGTAACAATGCCATGT

### 2.15 Hematoxylin and eosin (HE) staining and Masson staining

Lung tissues were fixed in 4% paraformaldehyde, dehydrated through an ethanol gradient, embedded in paraffin, and sectioned (4 μm). Sections were stained with HE and Masson’s trichrome, and then mounted with neutral resin. Morphological changes in pulmonary arteries and collagen deposition were examined using an optical microscope. Collagen fibers in Masson-stained sections were quantified using ImageJ.

### 2.16 Statistical analysis

Statistical analysis was conducted using SPSS version 21.0. Data were tested for normality and expressed as mean ± standard deviation (mean ± SD). Differences between the two groups were assessed by a two-tailed independent samples Student's t-test. Pearson’s correlation coefficient was used to examine the relationship between two variables. *p* < 0.05 was considered statistically significant.

## 3. Results

### 3.1 ssGSEA of immune infiltration

**Fig 2:**
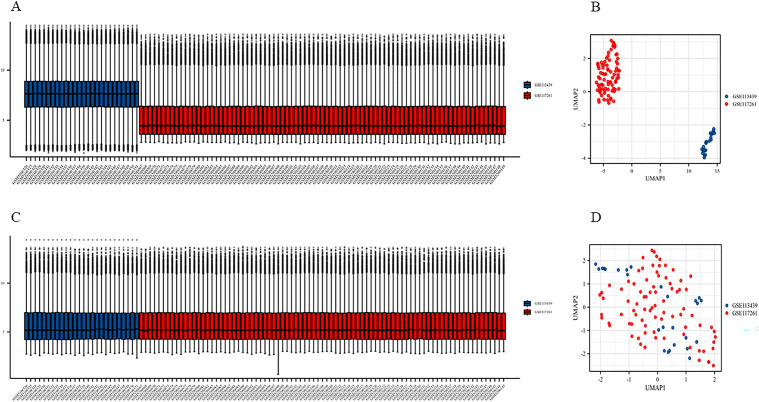
Assessment of batch effects and harmonization of integrated PAH transcriptomic datasets. **(A)** Boxplots of normalized gene-expression values across individual samples before batch-effect correction, colored by study origin (GSE113439, blue; GSE117261, red); **(B)** UMAP projection of uncorrected expression data, demonstrating dataset-driven separation of samples, indicating batch effects. Each dot represents one sample; **(C)** Boxplots of gene-expression distributions after batch-effect correction; **(D)** UMAP projection after batch-effect correction, showing improved inter-dataset integration and reduced dataset-driven clustering.

**Fig 3:**
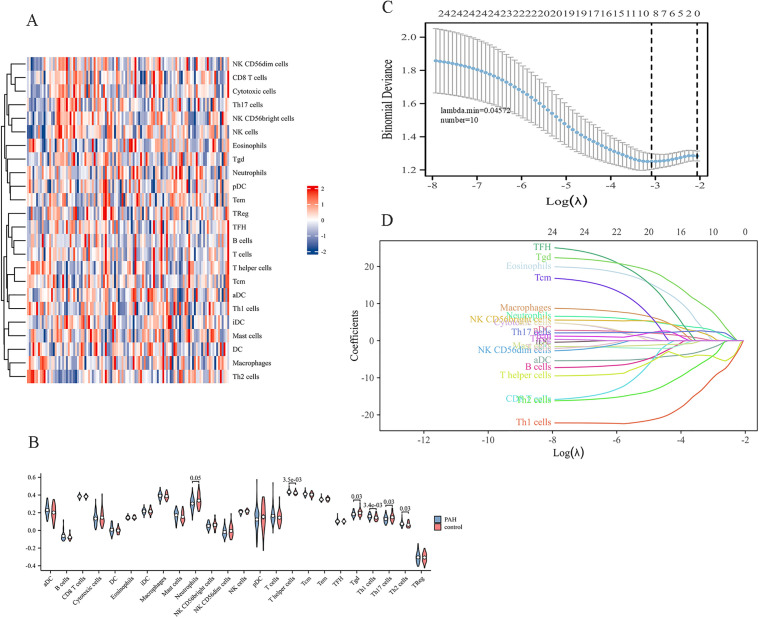
Immune-cell infiltration analysis in PAH and control lung tissues. **(A)** Heatmap illustrating the relative abundance of 24 immune-cell subsets across individual samples, as inferred by ssGSEA; **(B)** Violin plots showing differential comparison of immune-cell infiltration between PAH and control lung tissues using the Wilcoxon rank-sum test; **(C)** LASSO regression coefficient profiles of immune-cell subsets across varying penalty parameters (λ); **(D)** LASSO coefficient profiles of immune-cell subsets across varying values of λ, illustrating shrinkage and selection of immune-cell features associated with PAH classification.

Data from the GEO database (GSE113439 and GSE117261) were analyzed, comprising 73 PAH samples and 36 normal samples. According to the “ComBat” function from the R package “sva”, batch effects were corrected. Data before and after correction are shown in Fig 2A-2B and Fig 2C-2D, respectively, demonstrating successful batch effect removal and data normalization. The relative abundance of 24 immune cell subsets in all the samples is shown as a heatmap (Fig 3A). To identify immune cells that were not comparable between PAH and control lung tissues, we used a two-step approach: the Wilcoxon test followed by LASSO regression. Specifically, through the Wilcoxon test, violin plots for five immune cell types were generated, showing significant between-group differences (Fig 3B). Compared with normal controls, PAH tissues exhibited higher levels of T helper cells, Th1, and Th2 cells, whereas γδ T-cell (Tgd) and Th7 cells were relatively enriched in the control group (all *p* < 0.05). Consistently, LASSO regression (Fig 3C-3D) identified T helper cells and Th1 cells as the most informative immune features associated with PAH.

### 3.2 WGCNA

**Fig 4:**
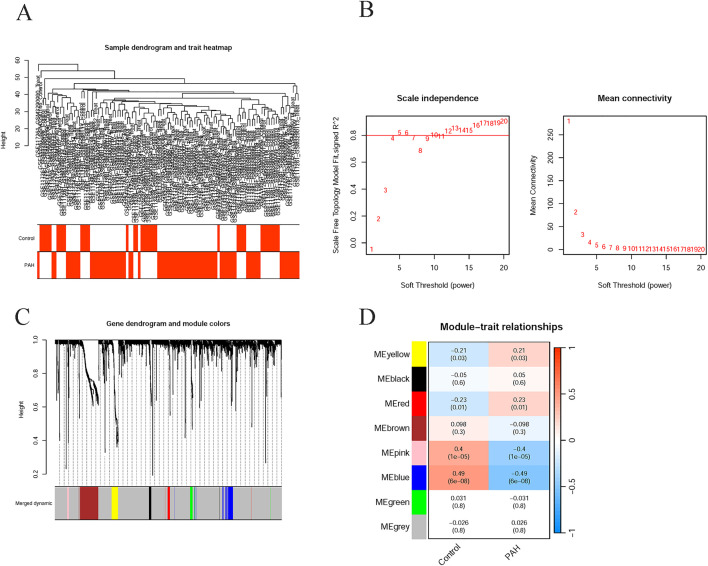
WGCNA of integrated PAH transcriptomic data. **(A)** Sample clustering dendrogram of the merged dataset with corresponding clinical trait annotation (Control vs. PAH), used to detect potential outliers prior to network construction; **(B)** Determination of the optimal soft-thresholding power for network construction; **(C)** Gene dendrogram and co-expression modules identified by dynamic tree cut (colored bars); **(D)** Heatmap of module-trait relationships, showing correlations between module eigengenes and clinical phenotype (Control vs. PAH). Correlation coefficients and corresponding *p*-values are displayed in each cell.

After merging the GSE113439 and GSE117261 datasets, all samples were included in WGCNA (Fig 4A). Based on the scale-free fit index and mean connectivity soft-threshold power 13 was selected as optimal (Fig 4B). By setting this parameter, the subsequent cut height was set to 0.25 to merge similar modules. Additionally, the TOM was constructed, and hierarchical clustering was performed to identify co-expression modules. A total of 8 different gene modules were identified, and each module was visualized as a different color (Fig 4C). The analysis of module-trait relationships, based on module eigengenes (defined as the first principal component of each gene module and representing its overall expression profile), revealed significant associations between specific modules and PAH. (Fig 4D).

### 3.3 Screening of DEGs and related functional analysis

**Fig 5:**
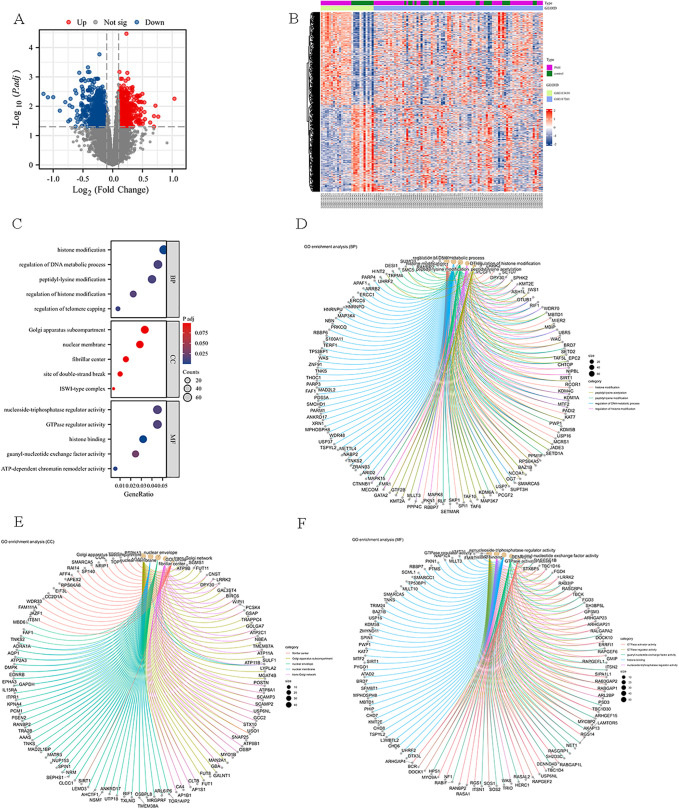
Identification of DEGs and GO enrichment analysis in PAH lung tissues. **(A)** Volcano plot of DEGs between PAH and control samples. Red: upregulated; blue: downregulated; and gray: non-significant; x-axis: log2 fold change; and y-axis: -log10 (adjusted *p* value); **(B)** Heatmap of DEGs across individual samples, illustrating distinct expression patterns between PAH and control groups; **(C)** Bubble plot of GO enrichment results (BP, CC, and MF categories); x-axis: gene ratio; bubble size: gene counts; **(D-F)** Circular network plots illustrating the relationships between enriched GO terms and associated genes for BP **(D)**, CC **(E)**, and MF **(F)**, respectively.

Differential expression analysis identified 1,511 DEGs in PAH samples compared to controls, including 730 upregulated and 781 downregulated genes. The DEGs were visualized and interpreted using heatmaps and volcano plots (Fig 5A-5B). Moreover, GO enrichment analysis revealed that biological processes (BP) related to histone modification and regulation of DNA metabolic processes were significantly enriched, suggesting epigenetic alterations in PAH. In the cellular components (CC), the results revealed the significant enrichment in the Golgi apparatus subcompartment and nuclear membrane. Meanwhile, regarding molecular functions (MF), nucleoside triphosphatase regulator activity and GTPase regulator activity were notably enriched. The top-five enriched terms in each category (p. adj < 0.05) are shown in bar plots (Fig 5C). Besides, other circular plots were plotted to graphically illustrate BP, CC and MF results (Fig 5D-5F).

### 3.4 Identification and validation of hub genes

**Fig 6:**
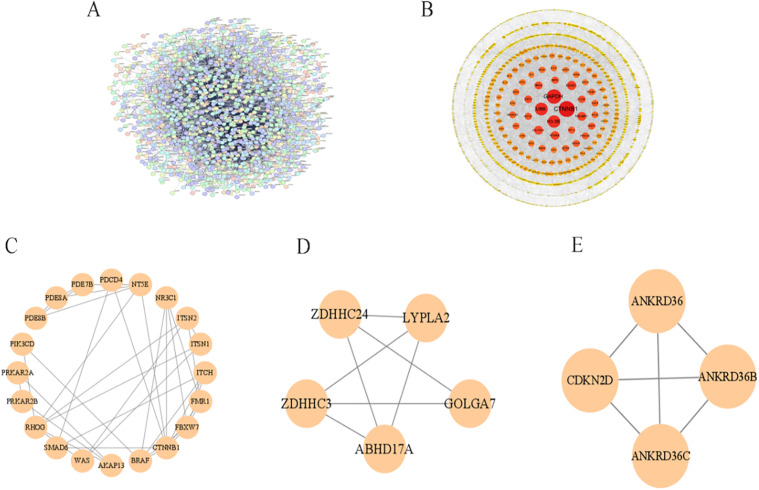
PPI network construction and module identification of DEGs. **(A)** Global PPI network of DEGs using the STRING database. Nodes: proteins; edges: predicted or experimentally validated interactions; **(B)** Visualization of the PPI network in Cytoscape software, highlighting hub genes and network topology; **(C-E)** Highly interconnected gene clusters identified using the MCODE algorithm, representing core functional modules within the PPI network.

**Fig 7:**
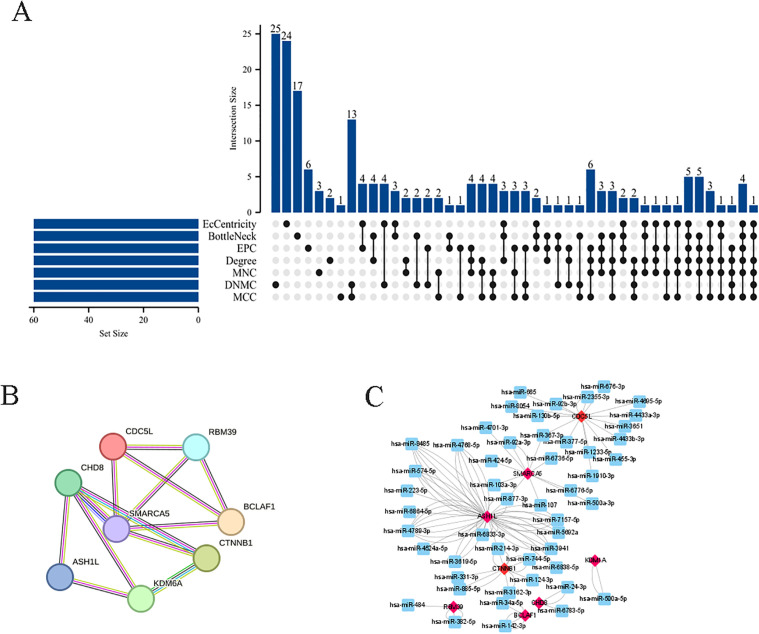
Hub gene identification and the co-expression network involving mRNA and target miRNA. **(A)** Workflow for screening hub genes; **(B)** PPI network (constructed using the STRING software) of the eight identified hub genes; **(C)** The mRNA-miRNA co-expression network.

**Fig 8:**
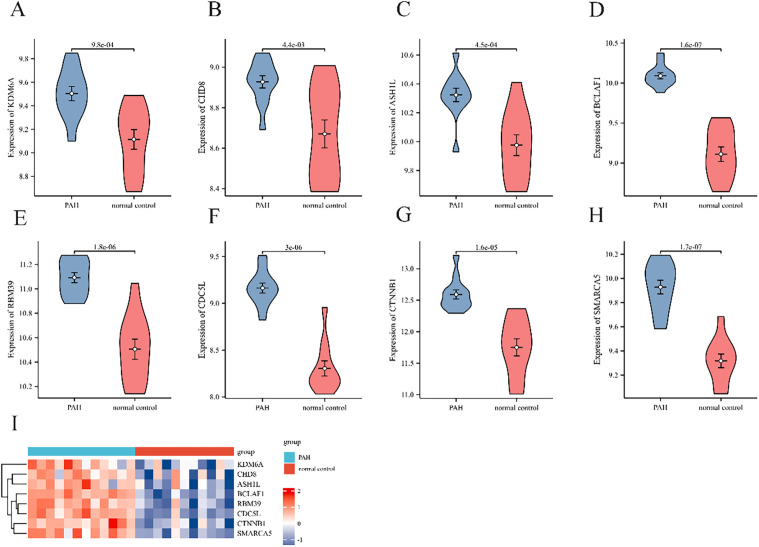
External validation of hub gene expression in the GSE53408 dataset. **(A-H)** Violin plots showing the expression levels of eight hub genes in PAH and control lung tissues from the independent validation cohort (GSE53408). Statistical significance between groups was assessed using the Wilcoxon rank-sum test; **(I)** Heatmap of hub gene expression, demonstrating distinct clustering between PAH and control groups.

A PPI network of DEGs was constructed by STRING, comprising 1,393 nodes and 6,949 edges (average node degree 9.98). Unconnected protein nodes were excluded (Fig 6A). The data were imported into Cytoscape for visualization, with node sizes adjusted according to betweenness centrality BC values (Fig 6B). Three gene cluster modules were identified by MCODE: Cluster 1 (20 edges; 37 nodes), Cluster 2 (5 edges; 7 nodes), and Cluster 3 (4 edges; 6 nodes) (Fig 6C-6E). Subsequently, the top 60 genes were filtered using seven algorithms with the R package “UpSet”, identifying 8 DEGs (Fig 7A): RBM39, BCLAF1, CDC5L, CTNNB1, SMARCA5, CHD8, ASH1L, and KDM6A. The PPI network of these genes was visualized in Fig 7B. Additionally, a co-expression network featuring eight genes was developed using Cytoscape (Fig 7C), which includes both mRNA and miRNA. Hub gene expression was validated using the dataset GSE53408. The analysis revealed that all eight hub genes were significantly upregulated in PAH samples compared with normal lung tissues (Fig 8A-8H). Additionally, a heatmap was generated to effectively visualize their expression patterns (Fig 8I).

### 3.5 Screening biomarkers of PAH

**Fig 9:**
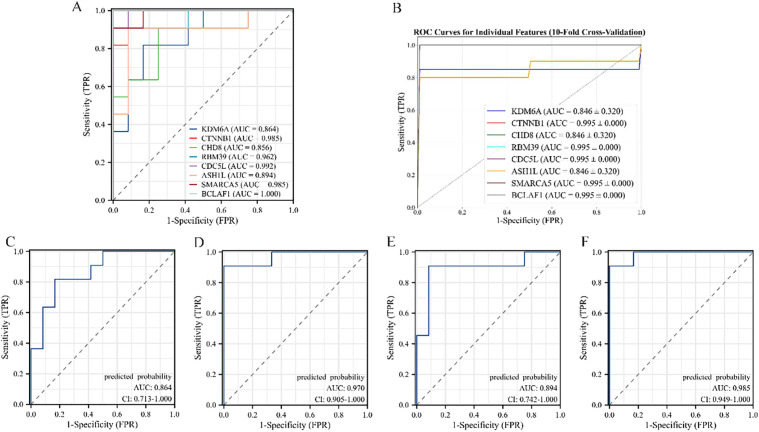
Diagnostic performance of hub genes in PAH lung tissues. **(A)** The diagnostic value of 8 hub genes in PAH was evaluated by the GSE53408 datasets; **(B)** Ten-fold cross-validation results; **(C)** The diagnostic value of KDM6A and CTNNB1 in PAH; **(D)** The diagnostic value of CHD8 and RBM391 in PAH; **(E)** The diagnostic value of CDC5L and ASH1L in PAH; **(F)** The diagnostic value of SMARCA5 and BCLAF1 in PAH.

The diagnostic value of eight hub genes was assessed using ROC analysis on the dataset GSE53408. It was found that the AUC typically ranged from 0.5 to 1, with values approaching 1 indicating better diagnostic performance for identifying PAH. Specifically, the following AUC values were observed: RBM39 (AUC = 0.962, CI = 0.883–1.000), BCLAF1 (AUC = 1.00, CI = 1.000–1.000), CDC5L (AUC = 0.992, CI = 0.971–1.000), CTNNB1 (AUC = 0.985, CI = 0.949–1.000), SMARCA5 (AUC = 0.985, CI = 0.949–1.000), CHD8 (AUC = 0.856, CI = 0.694–1.000), ASH1L (AUC = 0.894, CI = 0.742–1.000), and KDM6A (AUC = 0.864, CI = 0.713–1.000) (Fig 9A). To assess potential overfitting, ten-fold cross-validation was conducted (Fig 9B). Pairwise combinations of hub genes were also evaluated by ROC analysis. The combined AUC for KDM6A and CTNNB1 was 0.864 (CI = 0.713–1.000), for CHD8 and RBM39 was 0.970 (CI = 0.905–0.982), for CDC5L and ASH1L was 0.894 (CI = 0.742–1.000), and for SMARCA5 and BCLAF1 was 0.985 (CI = 0.949–1.000) (Fig 9C-9F). Among these combinations, the SMARCA5 and BCLAF1 pair demonstrated the best specificity.

### 3.6 Analysis of hub genes using machine learning

**Fig 10:**
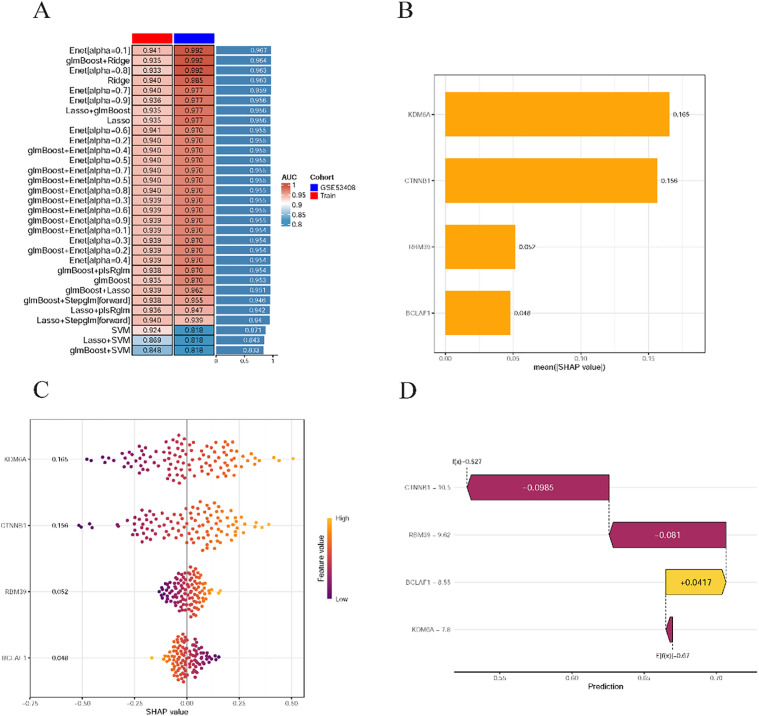
Machine learning-based prioritization and SHAP interpretability analysis of hub genes. **(A)** Heatmap comparing model performance across different machine learning algorithms, showing AUC values in the training cohort and external validation cohort (GSE53408); **(B)** Feature importance ranking based on mean absolute SHAP values, indicating the relative contribution of each hub gene to model prediction; **(C)** SHAP summary (beeswarm) plot illustrating the distribution of SHAP values for each gene across samples. Each point represents one sample, and color indicates feature expression level (high to low); **(D)** SHAP waterfall plot for a representative sample, demonstrating the individual contribution of each gene to the final prediction probability.

Through machine learning analysis of 8 hub genes, 113 predictive models were constructed to identify key genes. Among these, the Enet[alpha = 0.1] and glmBoost + Ridge model demonstrated superior performance and achieved high accuracy (Fig 10A). Four genes (KDM6A, CTNNB1, RBM39, and BCLAF1) were identified from hub genes according to machine learning (Fig 10B). Moreover, each gene's contribution to the model was quantified using SHAP values, with a larger value indicating greater contribution. It was found that KDM6A had the greatest contribution to the model (Fig 10B). SHAP dependence plots further clarified the directional influence of each gene in the model. Specifically, for KDM6A, CTNNB1, and RBM39, SHAP values increased as the Feature Value rose. In contrast, for BCLAF1, the SHAP value decreased as the Feature Value increased (Fig 10C). Furthermore, the comprehensive predicted value (f(x) = 0.527) was below the baseline of 0.67 (Fig 10D).

### 3.7 Correlation analysis between hub genes and immune cells

**Fig 11:**
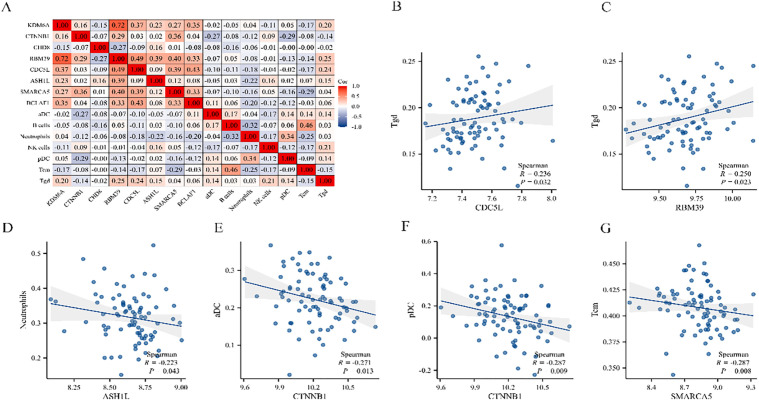
Correlation analysis between hub gene expression and immune-cell signatures in PAH lung tissues. **(A)** Heatmap of Spearman correlation coefficients between hub genes and inferred immune-cell subsets. Color intensity represents the strength and direction of correlation; **(B-C)** Positive correlations between CDC5L and RBM39 expression levels and Tgd signatures; **(D)** Negative correlation between ASH1L expression and neutrophil signatures; **(E-F)** Negative correlations between CTNNB1 expression and aDC and pDC; **(G)** Negative correlation between SMARCA5 expression and Tcm signatures. Spearman correlation coefficients (R) and corresponding *p* values are indicated in each panel.

A correlation analysis was conducted on eight hub genes, revealing their association with seven types of immune cells (Fig 11A). With a threshold of *p* < 0.05, immune cells and biomarkers with significant differential correlations were screened. The results indicated that CDC5L and RBM39 exhibited a positive correlation with Tgd cells (Fig 11B-11C), while ASH1L was negatively correlated with neutrophils (Fig 11D). Furthermore, CTNNB1 expression was negatively correlated with both activated dendritic cells (aDC) and plasmacytoid dendritic cells (pDC) (Fig 11E-11F). These findings suggested that higher CTNNB1 expression was associated with reduced predicted abundance and/or activation of the related DC subsets. Besides, central memory T cells (Tcm) were negatively related to SMARCA5 (Fig 11G), revealing that increased SMARCA5 levels were co-morbid with low Tcm representation and can consequently be linked to T-cell memory program maintenance or remodeling. These correlations were based on inferred immune cell signatures from transcriptomic data. Direct quantification of immune cell populations (e.g., flow cytometry or immunostaining) is warranted in future studies.

### 3.8 Validation of biomarkers in PAH Mice models

**Fig 12:**
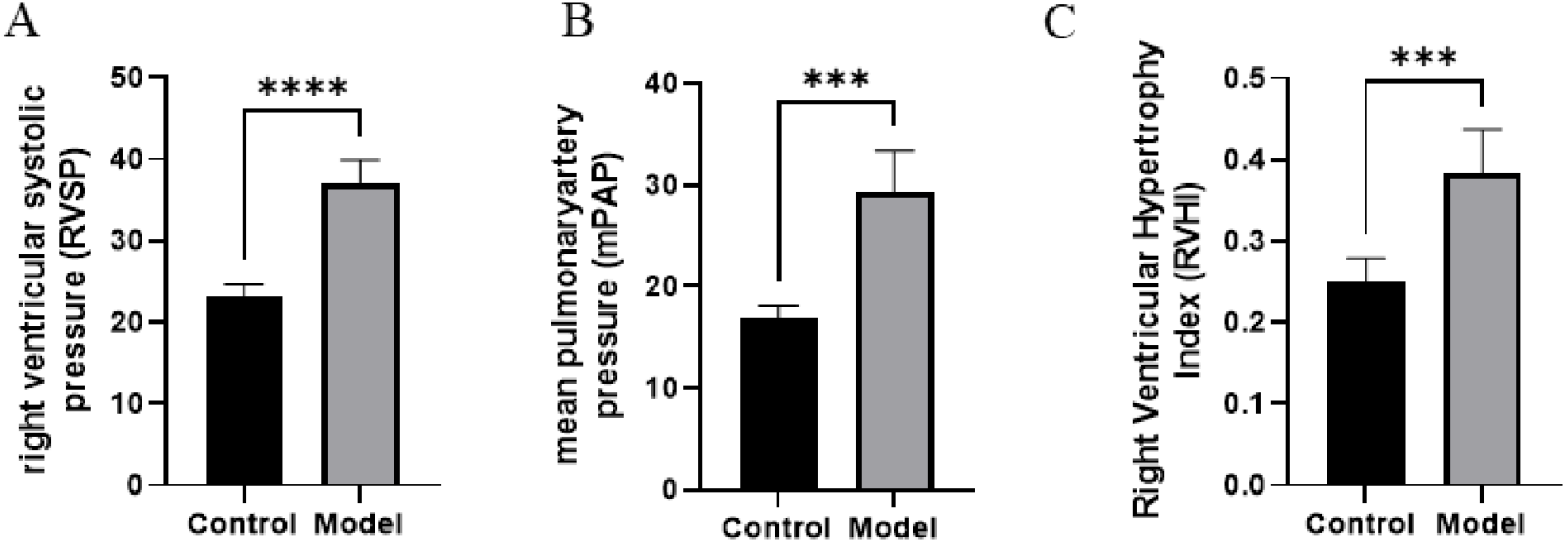
Hemodynamic validation of the PAH model. **(A)** Right ventricular systolic pressure (RVSP); **(B)** Mean pulmonary artery pressure (mPAP); **(C)** Right ventricular hypertrophy index (RVHI) in control and model mice. Data are presented as mean ± SD. Statistical comparisons between groups were performed using an unpaired Student’s t-test. *p < 0.05, **p < 0.01, ***p < 0.001, ****p < 0.0001, ns, no significant difference.

**Fig 13:**
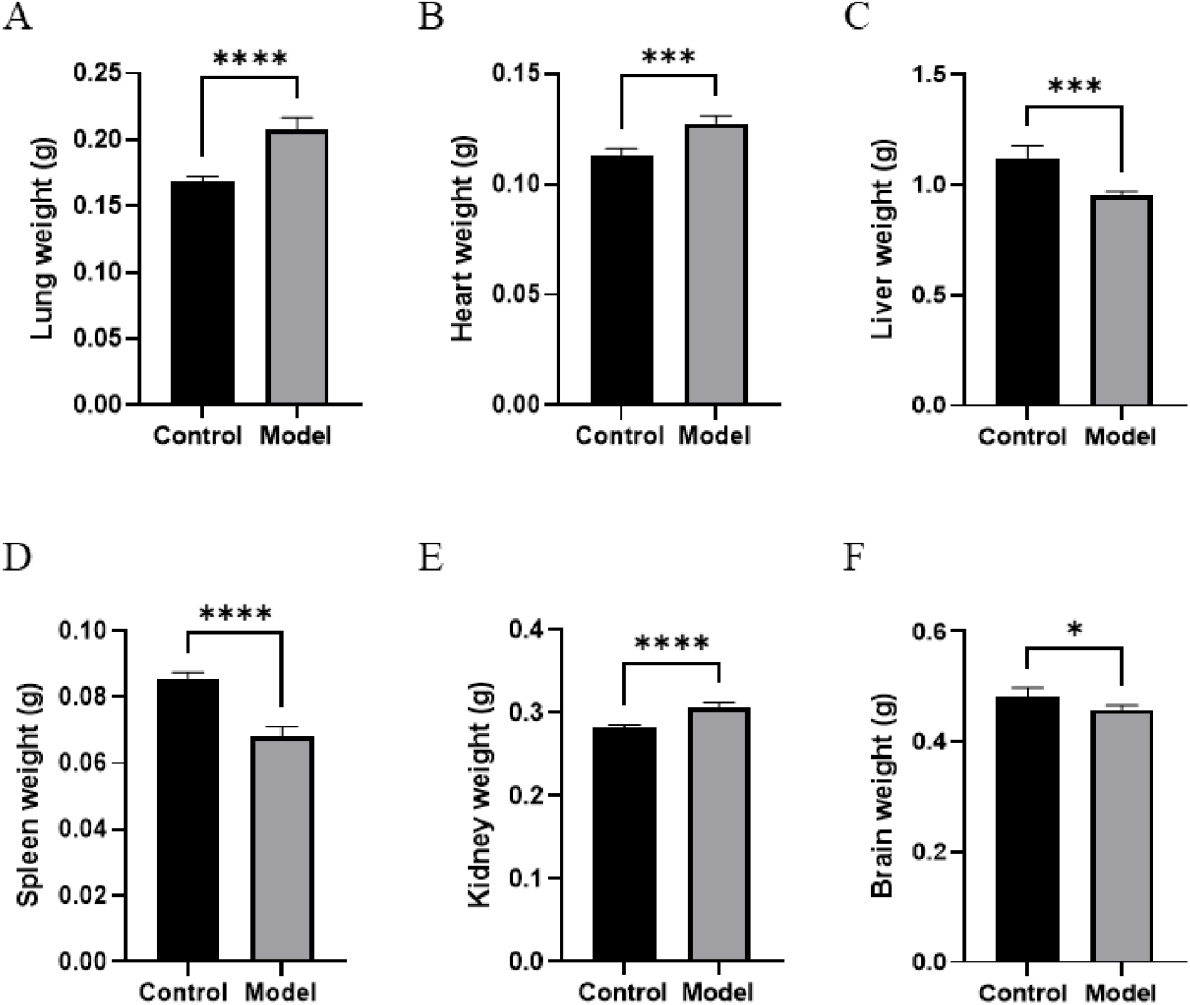
Comparison of organ weights between control and PAH model mice. **(A-F)** Absolute organ weights of lung **(A)**, heart **(B)**, liver **(C)**, spleen **(D)**, kidney **(E)**, and brain (F) in control and model groups. Data are presented as mean ± SD. Statistical comparisons between groups were performed using an unpaired Student’s t-test. *p < 0.05, **p < 0.01, ***p < 0.001, ****p < 0.0001, ns, no significant difference.

To further investigate the expression and localization of hub genes in PAH, we constructed a mouse model of PAH, with normally raised mice serving as the control group. After model construction, tissues were harvested from both groups for analysis. As shown in Fig 12, compared with those in the control group, mice in the model group exhibited a significant elevation in RVSP, mPAP, and RVHI (*p* < 0.05). These findings collectively indicated successful establishment of the model. Subsequently, it was revealed that there was a significant difference in the size of certain organs between the two groups. Specifically, the lung, heart, and kidney tissues from the PAH group were notably larger compared to the corresponding tissues in the control group (Fig 13). Conversely, the tissues of the liver, spleen, and brain in the control group were markedly larger than those of the same organs in the PAH group (Fig 13). This discrepancy suggested differential organ-specific effects of PAH pathophysiology. The RVHI was significantly increased in PAH mice, reflecting more severe pulmonary lesions driven by inflammatory exudation and tissue remodeling.

**Fig 14:**
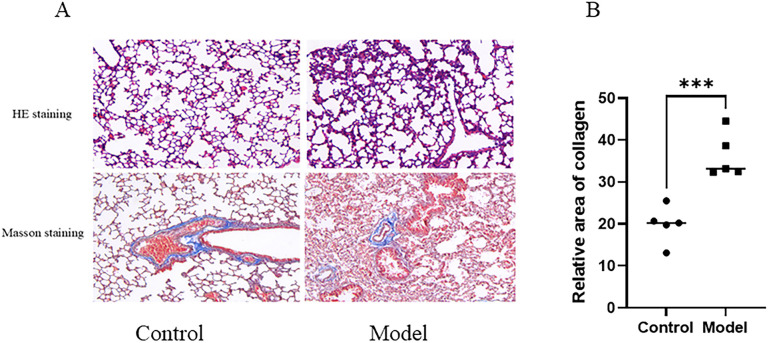
Histological assessment of lung tissues in control and PAH model mice. **(A)** Representative HE staining and Masson’s trichrome staining images of lung sections from control and PAH model groups. Scale bar = 100 μm; **(B)** Quantification of collagen area fraction based on Masson staining. Data are presented as mean ± SD. Statistical comparison was performed using an unpaired Student’s t-test. ***p < 0.001.

HE staining showed that lung tissues from control mice displayed preserved alveolar architecture and orderly structural organization. In contrast, lung tissues from the PAH model group exhibited disrupted tissue architecture, thickening of vascular walls, and increased cellularity within the perivascular regions (Fig 14A). These morphological alterations are consistent with vascular remodeling observed in experimental pulmonary hypertension. Masson staining further demonstrated increased collagen deposition in the model group compared to controls, indicating enhanced extracellular matrix accumulation and vascular remodeling (Fig 14A-14B).

**Fig 15:**
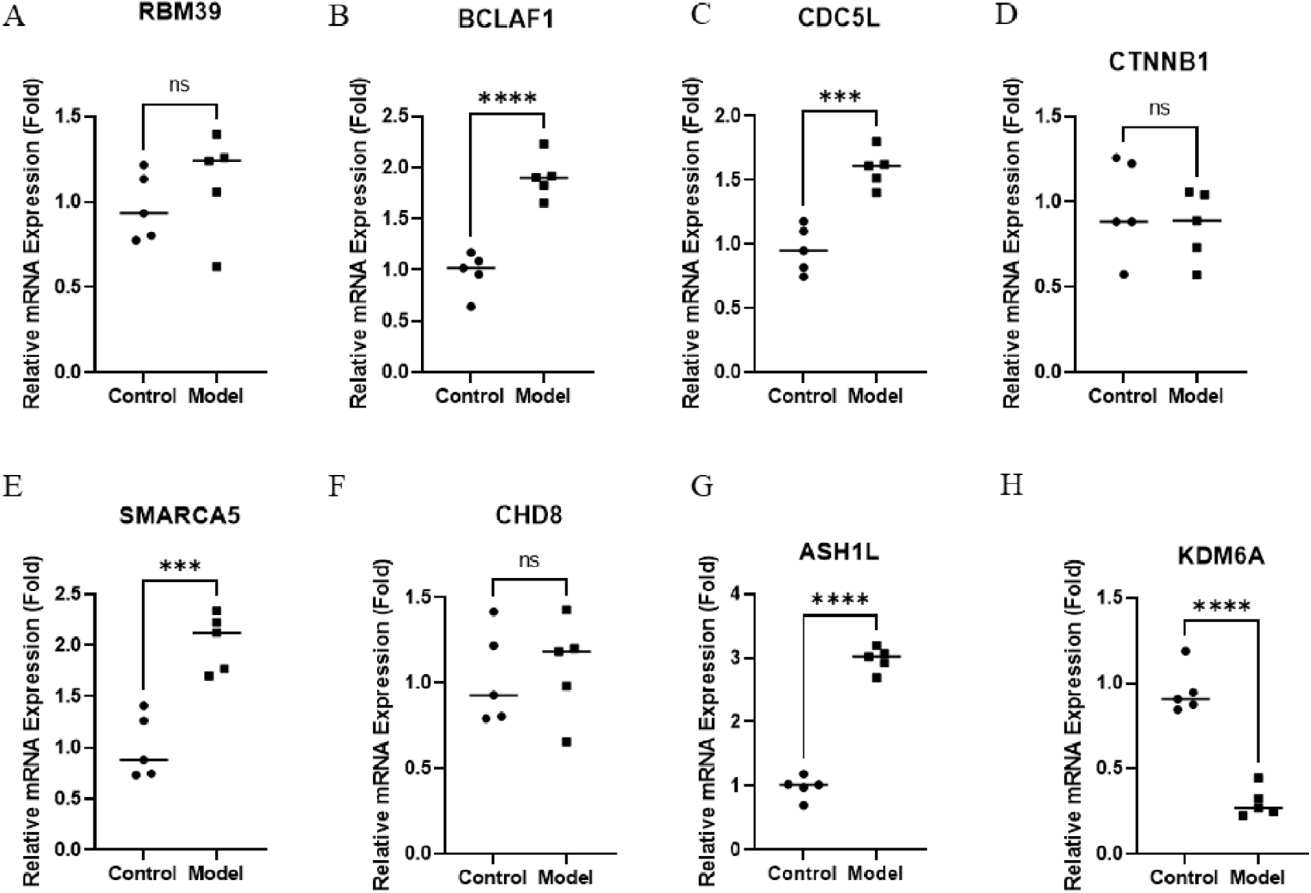
Validation of hub gene expression in lung tissues from control and PAH model mice by qRT-PCR. A-H. Relative mRNA expression levels of the eight hub genes (RBM39, BCLAF1, CDC5L, CTNNB1, SMARCA5, CHD8, ASH1L, and KDM6A) were measured in lung tissues from control and SuHx-induced PAH mice using qRT-PCR. Data are presented as mean ± SD. Statistical comparisons between groups were performed using an unpaired Student’s t-test. ns, not significant; ***p < 0.001; ****p < 0.0001.

qRT-PCR analysis revealed that the expression levels of BCLAF1, CDC5L, SMARCA5, and ASH1L were significantly elevated in lung tissues from the PAH model group relative to controls (Fig 15). These findings supported the transcriptome-based observations from human PAH datasets and validated the dysregulation of selected hub genes in a PAH-relevant *in-vivo* model. However, immune-cell populations were not quantitatively assessed in this model. Hence, the observed histological changes should not be interpreted as direct evidence of specific immune-cell infiltration.

## 4. Discussion

Studies have shown that PAH lungs have an immune imbalance, characterized by changes in both myeloid and lymphoid signatures accompanied by enrichment of pro-inflammatory signaling pathways [27–31]. However, few studies have linked these immune infiltration patterns to hub genes across independent PAH cohorts. In the present study, we systematically combined immune-cell inference, co-expression network analysis, multi-algorithm hub-gene screening, and predictive modeling to identify immune-related genes linked to PAH. This approach highlights genes that may play key roles in both immunity and disease diagnosis, and provides a structured basis for downstream mechanistic studies.

The immune infiltration analysis suggested increased T helper-associated signatures in PAH lungs, including Th1- and Th2-related signals. Prior studies have demonstrated that Th1, Th2, and Th17 subsets contribute to pulmonary vascular inflammation and remodeling through cytokine-mediated effects on endothelial and smooth muscle cells [28–34]. For example, Th17-associated cytokines may promote pulmonary arterial smooth muscle cell (PASMC) proliferation and survival [28,29]. However, Th1-derived inflammatory mediators can amplify vascular inflammation [30,31]. Th2-related signaling has also been implicated in pulmonary vascular muscularization and remodeling [32,33]. In this context, our results supported a PAH immune landscape characterized by altered T-cell–associated transcriptomic signatures. Importantly, these findings are based on computational inference from transcriptomic data, and therefore warrant direct validation in lung tissues.

The present study identified eight hub genes through integrative screening and validated their expression patterns in an independent dataset (GSE53408). ROC analysis indicated that BCLAF1, alone or combined with SMARCA5, demonstrated favorable diagnostic performance. Considering the limited size of the external validation cohort, cross-validation was performed to provide more conservative estimates of the AUC.

Among the prioritized genes, BCLAF1 has been implicated in apoptosis regulation and hypoxia-associated transcriptional pathways [35]. As has been evidenced previously, BCLAF1 interacts with anti-apoptotic Bcl2 family members [36] and may participate in smooth muscle lineage regulation [37]. In addition, BCLAF1 has been reported to interact with HIF-1α and NF-κB signaling pathways in other disease contexts [38–41]; both pathways are relevant to hypoxia-driven vascular remodeling and inflammatory signaling. Despite the biological plausibility of these observations, whether BCLAF1 directly modulates pulmonary vascular remodeling or immune responses in PAH requires functional validation.

SMARCA5 encodes the chromatin remodeling protein SNF2H and is involved in transcriptional regulation, endothelial function, and angiogenic responses [42]. Chromatin remodeling factors have increasingly been recognized as modulators of vascular homeostasis and inflammatory gene programs. In the context of PAH, dysregulated chromatin-associated regulators (such as SMARCA5) may contribute to altered endothelial and vascular cell phenotypes, though direct evidence in PAH remains limited. Accordingly, we focused on mechanisms related to immune remodeling.

Correlation analysis further suggested that CDC5L and RBM39 were positively associated with inferred Tgd signatures, whereas ASH1L showed an inverse association with neutrophil signatures. CTNNB1 was inversely correlated with activated and plasmacytoid DC signatures, and SMARCA5 was inversely associated with Tcm signatures. Accumulating studies have demonstrated that T lymphocytes, regulatory T cells (Tregs), neutrophils, and DCs are implicated in PAH pathobiology [8,43–47]. For example, Tregs may attenuate inflammatory signaling and modulate Akt/ERK pathways in pulmonary vascular remodeling [43,48]. Neutrophil elastase has been shown to contribute to extracellular matrix remodeling in PAH models [44,45]. Nevertheless, these findings are based on transcriptomic associations and do not provide direct evidence of immune-cell regulation.

To validate our computational findings, we examined hub-gene expression in a PAH-relevant experimental model (Su5416 combined with hypoxia). In this model, BCLAF1, CDC5L, SMARCA5, and ASH1L were significantly upregulated in lung tissues. Importantly, hemodynamic measurements (RVSP, mPAP, and RVHI) were incorporated to confirm the presence and severity of pulmonary hypertension, strengthening validation beyond organ weight or histological changes alone. Although the SuHx model reproduces key features of pulmonary hypertension, it does not fully replicate human complex plexiform vasculopathy, a limitation to consider when interpreting translational relevance.

This study identified hub genes that may serve as candidate biomarkers for PAH diagnosis and stratification. In the future, these findings should be validated in larger PAH cohorts, and protein-level expression should be evaluated using cell-resolved approaches such as multiplex immunostaining or spatial transcriptomics. Functional studies in pulmonary vascular and immune cell populations are needed to establish causality. If validated, these hub genes may inform biomarker development and potentially guide pathway-oriented therapeutic strategies.

Nevertheless, this study has several limitations. First, the external validation cohort (GSE53408) was relatively small; single-gene AUC estimates may be unstable, warranting validation in larger independent cohorts and prospective studies. Second, although the SuHx model is widely used as a PAH-relevant platform, it does not fully recapitulate the complex vascular lesions of human PAH. Third, immune-cell populations were not quantitatively profiled in the murine model, and hub gene–immune cell relationships were not directly tested *in vivo*. Therefore, these immune-related findings are preliminary and require further experimental validation.

Collectively, the present findings establish an immune-integrated transcriptomic framework for prioritizing PAH biomarkers across multiple cohorts. While experimental validation is still needed, this strategy provides a scalable model for integrating immune inference with network biology in complex vascular diseases. Future studies combining longitudinal patient cohorts, spatial transcriptomics, and functional perturbation models will be essential to determine whether these hub genes represent merely correlates of immune remodeling or active drivers of PAH progression.

## 5. Conclusion

This study identifies immune-associated hub genes in PAH through integrative multi-dataset analysis, incorporating immune infiltration profiling and network-based prioritization. *In-vivo* validation in a PAH mouse model further supports the robustness of the identified candidates. These findings suggest that these genes may serve as potential diagnostic biomarkers and help explain immune-related mechanisms in PAH pathogenesis. Nevertheless, the exact functional roles of these hub genes need to be investigated through future mechanistic studies.
